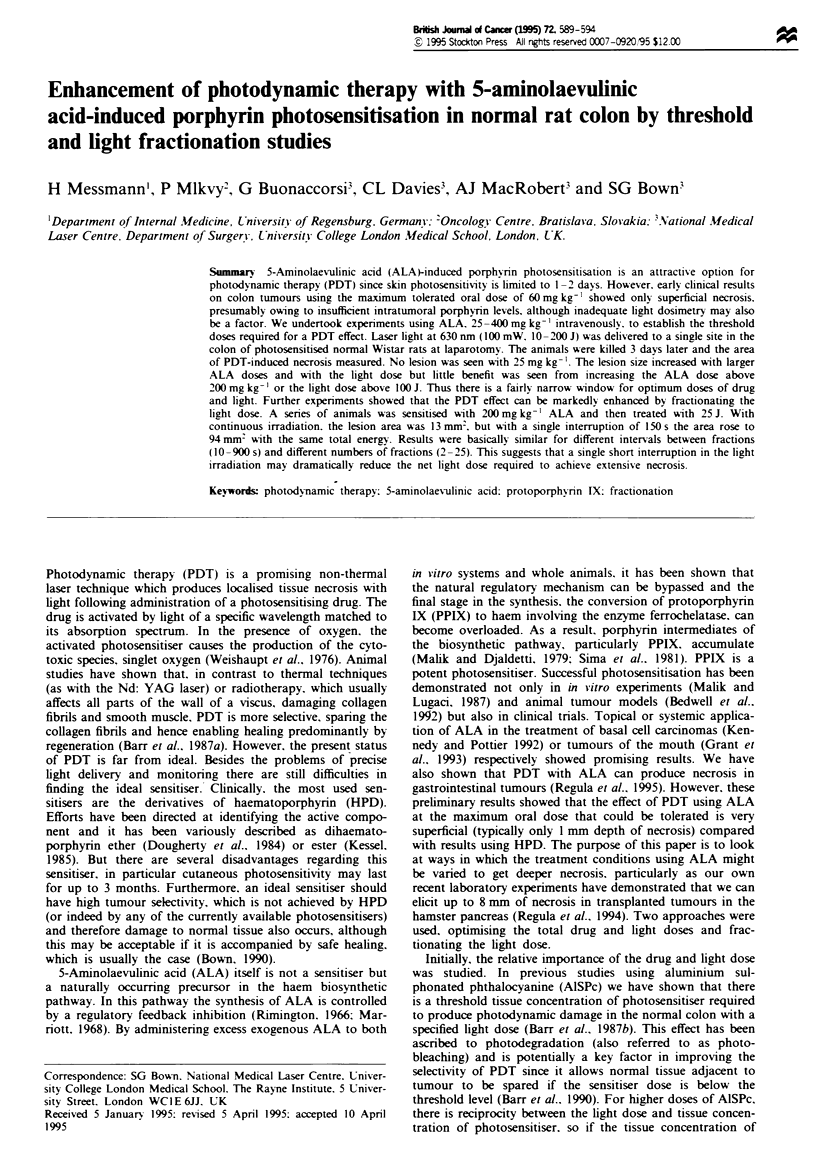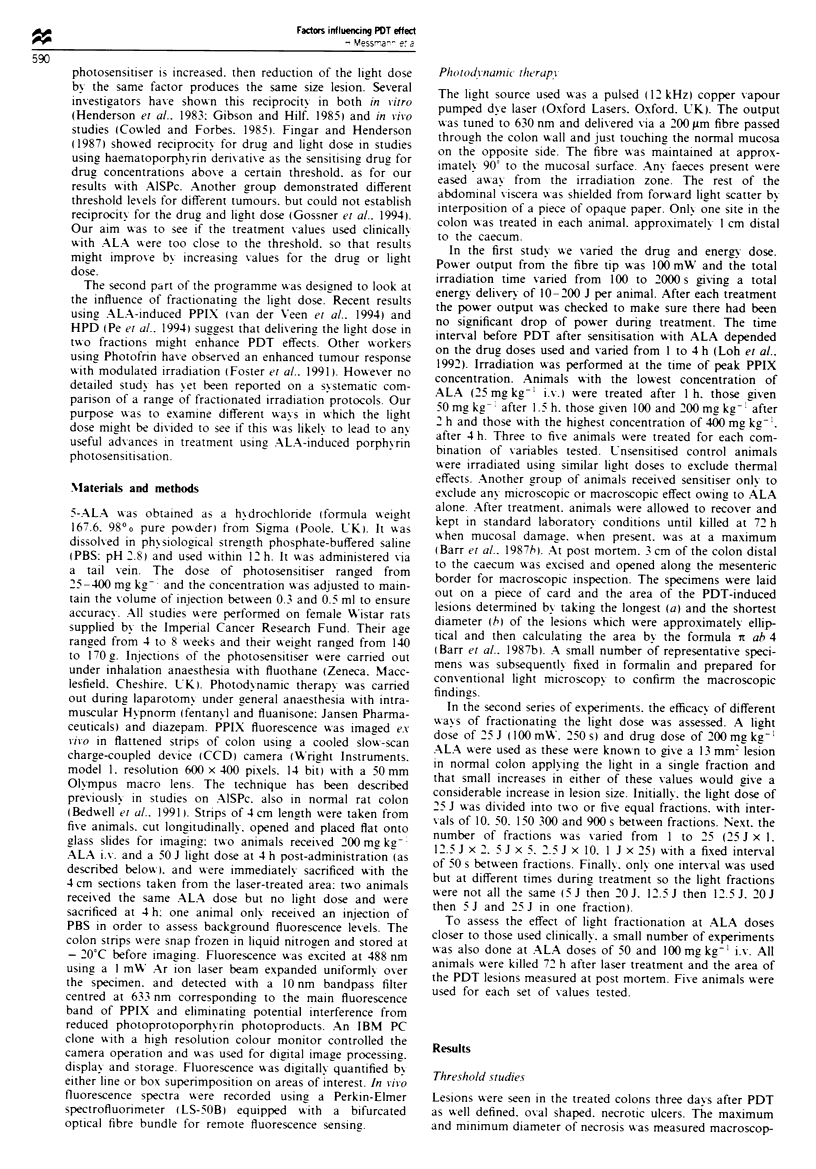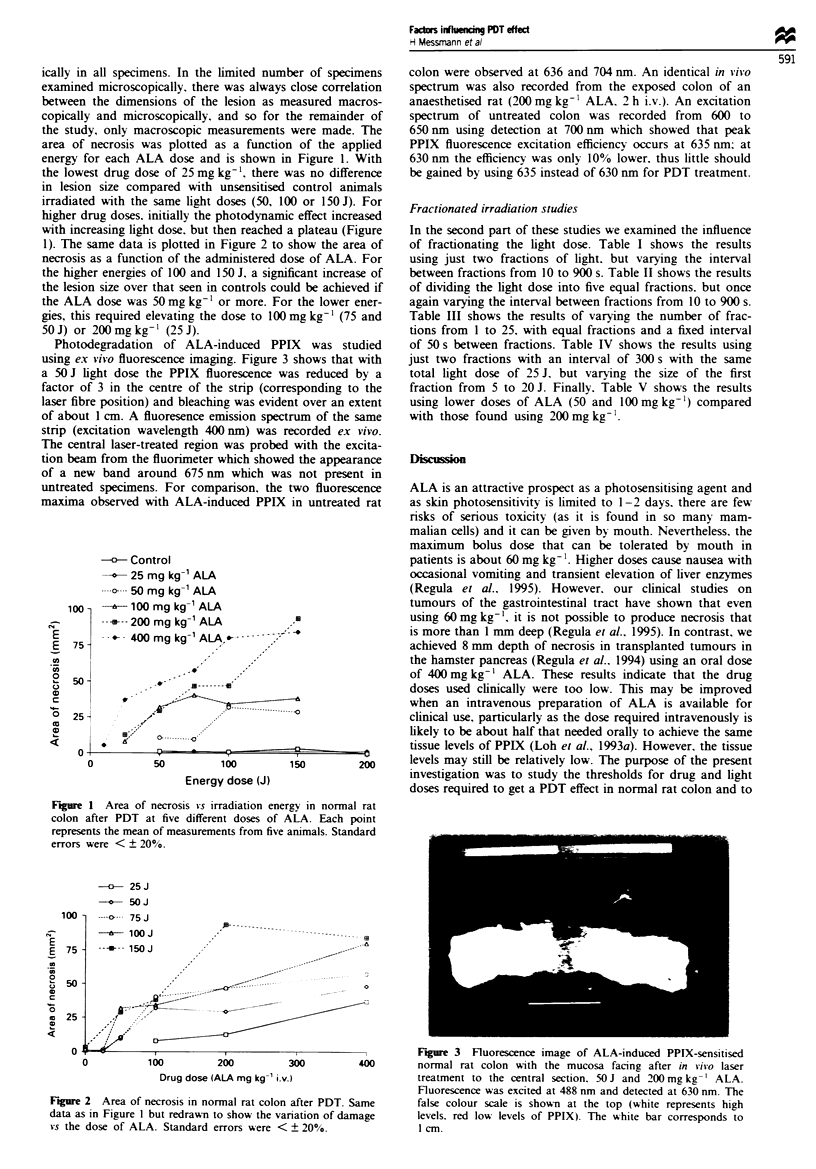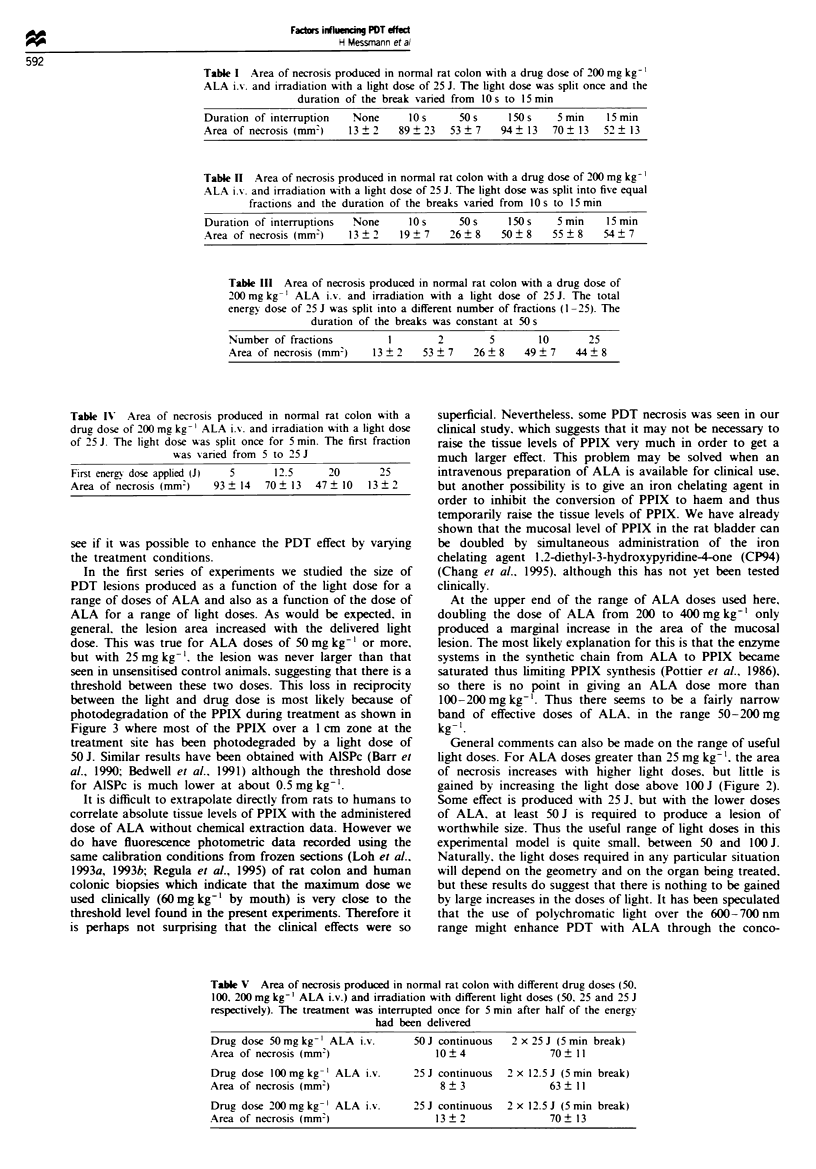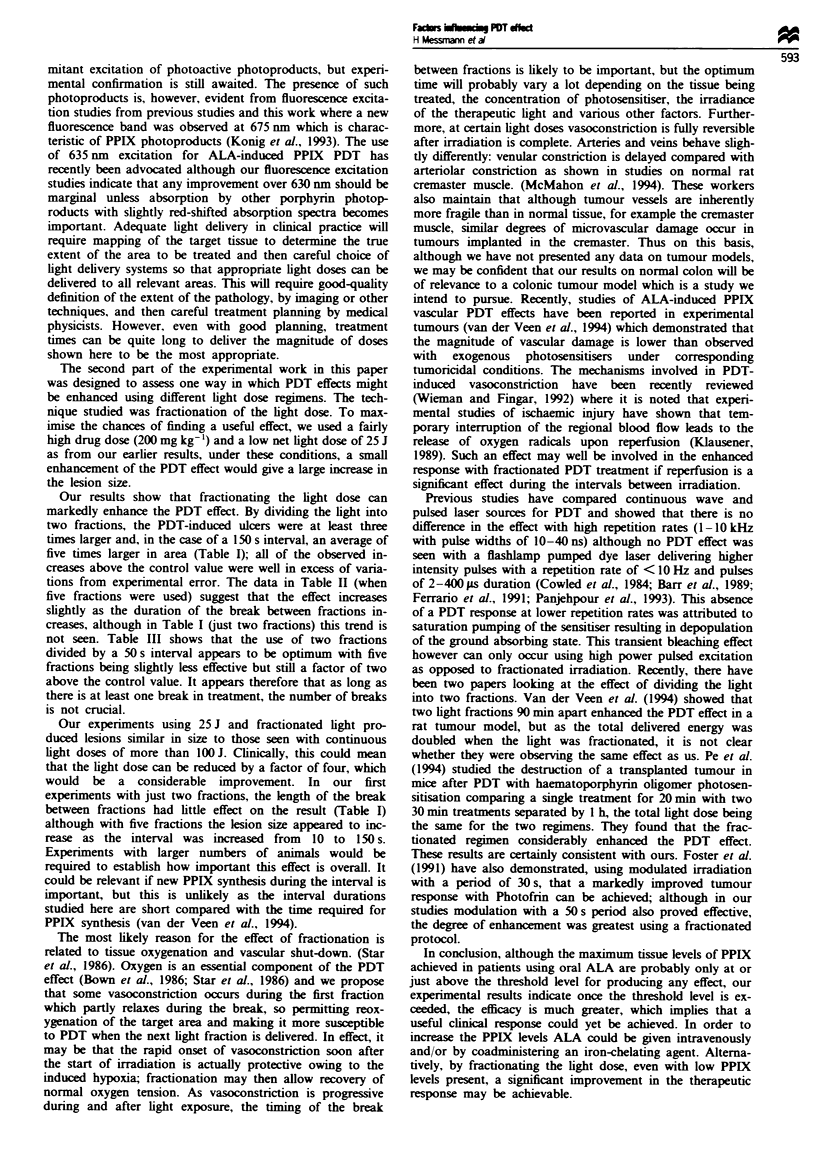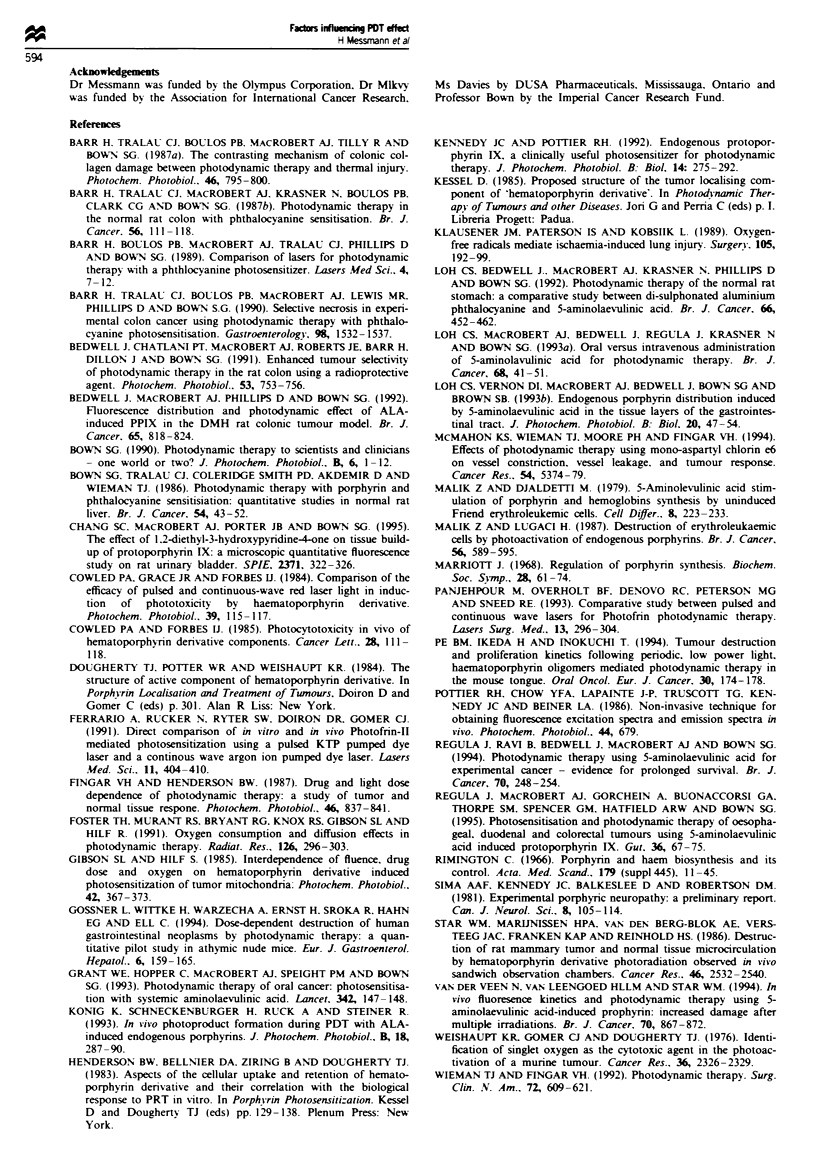# Enhancement of photodynamic therapy with 5-aminolaevulinic acid-induced porphyrin photosensitisation in normal rat colon by threshold and light fractionation studies.

**DOI:** 10.1038/bjc.1995.378

**Published:** 1995-09

**Authors:** H. Messmann, P. Mlkvy, G. Buonaccorsi, C. L. Davies, A. J. MacRobert, S. G. Bown

**Affiliations:** Department of Internal Medicine, University of Regensburg, Germany.

## Abstract

**Images:**


British Journal of Cancer (1995) 72, 589-594

© 1995 Stockton Press All rights reserved 0007-0920/95 $12.00

### Enhancement of photodynamic therapy with 5-aminolaevulinic acid-induced porphyrin photosensitisation in normal rat colon by threshold and light fractionation studies

H Messmann[1], P Mlkvy[2], G Buonaccorsi[3], CL Davies[3], AJ MacRobert[3] and SG Bown[3]

[1]*Department of Internal Medicine, University of Regensburg, Germany;* [2]*Oncology Centre, Bratislava, Slovakia;* [3]*National Medical Laser Centre, Department of Surgery, University College London Medical School, London, UK.*

**Summary** 5-Aminolaevulinic acid (ALA)-induced porphyrin photosensitisation is an attractive option for photodynamic therapy (PDT) since skin photosensitivity is limited to 1-2 days. However, early clinical results on colon tumours using the maximum tolerated oral dose of 60 mg $kg^{-1}$ showed only superficial necrosis, presumably owing to insufficient intratumoral porphyrin levels, although inadequate light dosimetry may also be a factor. We undertook experiments using ALA, 25-400 mg $kg^{-1}$ intravenously, to establish the threshold doses required for a PDT effect. Laser light at 630 nm (100 mW, 10-200 J) was delivered to a single site in the colon of photosensitised normal Wistar rats at laparotomy. The animals were killed 3 days later and the area of PDT-induced necrosis measured. No lesion was seen with 25 mg $kg^{-1}$. The lesion size increased with larger ALA doses and with the light dose but little benefit was seen from increasing the ALA dose above 200 mg $kg^{-1}$ or the light dose above 100 J. Thus there is a fairly narrow window for optimum doses of drug and light. Further experiments showed that the PDT effect can be markedly enhanced by fractionating the light dose. A series of animals was sensitised with 200 mg $kg^{-1}$ ALA and then treated with 25 J. With continuous irradiation, the lesion area was 13 $mm^2$, but with a single interruption of 150 s the area rose to 94 $mm^2$ with the same total energy. Results were basically similar for different intervals between fractions (10-900 s) and different numbers of fractions (2-25). This suggests that a single short interruption in the light irradiation may dramatically reduce the net light dose required to achieve extensive necrosis.

**Keywords:** photodynamic therapy; 5-aminolaevulinic acid; protoporphyrin IX; fractionation

Photodynamic therapy (PDT) is a promising non-thermal laser technique which produces localised tissue necrosis with light following administration of a photosensitising drug. The drug is activated by light of a specific wavelength matched to its absorption spectrum. In the presence of oxygen, the activated photosensitiser causes the production of the cytotoxic species, singlet oxygen (Weishaupt *et al.*, 1976). Animal studies have shown that, in contrast to thermal techniques (as with the Nd: YAG laser) or radiotherapy, which usually affects all parts of the wall of a viscus, damaging collagen fibrils and smooth muscle, PDT is more selective, sparing the collagen fibrils and hence enabling healing predominantly by regeneration (Barr *et al.*, 1987*a*). However, the present status of PDT is far from ideal. Besides the problems of precise light delivery and monitoring there are still difficulties in finding the ideal sensitiser. Clinically, the most used sensitisers are the derivatives of haematoporphyrin (HPD). Efforts have been directed at identifying the active component and it has been variously described as dihaematoporphyrin ether (Dougherty *et al.*, 1984) or ester (Kessel, 1985). But there are several disadvantages regarding this sensitiser, in particular cutaneous photosensitivity may last for up to 3 months. Furthermore, an ideal sensitiser should have high tumour selectivity, which is not achieved by HPD (or indeed by any of the currently available photosensitisers) and therefore damage to normal tissue also occurs, although this may be acceptable if it is accompanied by safe healing, which is usually the case (Bown, 1990).

5-Aminolaevulinic acid (ALA) itself is not a sensitiser but a naturally occurring precursor in the haem biosynthetic pathway. In this pathway the synthesis of ALA is controlled by a regulatory feedback inhibition (Rimington, 1966; Marriott, 1968). By administering excess exogenous ALA to both *in vitro* systems and whole animals, it has been shown that the natural regulatory mechanism can be bypassed and the final stage in the synthesis, the conversion of protoporphyrin IX (PPIX) to haem involving the enzyme ferrochelatase, can become overloaded. As a result, porphyrin intermediates of the biosynthetic pathway, particularly PPIX, accumulate (Malik and Djaldetti, 1979; Sima *et al.*, 1981). PPIX is a potent photosensitiser. Successful photosensitisation has been demonstrated not only in *in vitro* experiments (Malik and Lugaci, 1987) and animal tumour models (Bedwell *et al.*, 1992) but also in clinical trials. Topical or systemic application of ALA in the treatment of basal cell carcinomas (Kennedy and Pottier 1992) or tumours of the mouth (Grant *et al.*, 1993) respectively showed promising results. We have also shown that PDT with ALA can produce necrosis in gastrointestinal tumours (Regula *et al.*, 1995). However, these preliminary results showed that the effect of PDT using ALA at the maximum oral dose that could be tolerated is very superficial (typically only 1 mm depth of necrosis) compared with results using HPD. The purpose of this paper is to look at ways in which the treatment conditions using ALA might be varied to get deeper necrosis, particularly as our own recent laboratory experiments have demonstrated that we can elicit up to 8 mm of necrosis in transplanted tumours in the hamster pancreas (Regula *et al.*, 1994). Two approaches were used, optimising the total drug and light doses and fractionating the light dose.

Initially, the relative importance of the drug and light dose was studied. In previous studies using aluminium sulphonated phthalocyanine (AlSPc) we have shown that there is a threshold tissue concentration of photosensitiser required to produce photodynamic damage in the normal colon with a specified light dose (Barr *et al.*, 1987*b*). This effect has been ascribed to photodegradation (also referred to as photobleaching) and is potentially a key factor in improving the selectivity of PDT since it allows normal tissue adjacent to tumour to be spared if the sensitiser dose is below the threshold level (Barr *et al.*, 1990). For higher doses of AlSPc, there is reciprocity between the light dose and tissue concentration of photosensitiser, so if the tissue concentration of

Correspondence: SG Bown, National Medical Laser Centre, University College London Medical School, The Rayne Institute, 5 University Street, London WC1E 6JJ, UK
Received 5 January 1995; revised 5 April 1995; accepted 10 April 1995

photosensitiser is increased, then reduction of the light dose by the same factor produces the same size lesion. Several investigators have shown this reciprocity in both *in vitro* (Henderson *et al.*, 1983; Gibson and Hilf, 1985) and *in vivo* studies (Cowled and Forbes, 1985). Fingar and Henderson (1987) showed reciprocity for drug and light dose in studies using haematoporphyrin derivative as the sensitising drug for drug concentrations above a certain threshold, as for our results with AlSPc. Another group demonstrated different threshold levels for different tumours, but could not establish reciprocity for the drug and light dose (Gossner *et al.*, 1994). Our aim was to see if the treatment values used clinically with ALA were too close to the threshold, so that results might improve by increasing values for the drug or light dose.

The second part of the programme was designed to look at the influence of fractionating the light dose. Recent results using ALA-induced PPIX (van der Veen *et al.*, 1994) and HPD (Pe *et al.*, 1994) suggest that delivering the light dose in two fractions might enhance PDT effects. Other workers using Photofrin have observed an enhanced tumour response with modulated irradiation (Foster *et al.*, 1991). However no detailed study has yet been reported on a systematic comparison of a range of fractionated irradiation protocols. Our purpose was to examine different ways in which the light dose might be divided to see if this was likely to lead to any useful advances in treatment using ALA-induced porphyrin photosensitisation.

### Materials and methods

5-ALA was obtained as a hydrochloride (formula weight 167.6, 98% pure powder) from Sigma (Poole, UK). It was dissolved in physiological strength phosphate-buffered saline (PBS; pH 2.8) and used within 12 h. It was administered via a tail vein. The dose of photosensitiser ranged from 25–400 mg $kg^{-1}$ and the concentration was adjusted to maintain the volume of injection between 0.3 and 0.5 ml to ensure accuracy. All studies were performed on female Wistar rats supplied by the Imperial Cancer Research Fund. Their age ranged from 4 to 8 weeks and their weight ranged from 140 to 170 g. Injections of the photosensitiser were carried out under inhalation anaesthesia with fluothane (Zeneca, Macclesfield, Cheshire, UK). Photodynamic therapy was carried out during laparotomy under general anaesthesia with intramuscular Hypnorm (fentanyl and fluanisone; Jansen Pharmaceuticals) and diazepam. PPIX fluorescence was imaged *ex vivo* in flattened strips of colon using a cooled slow-scan charge-coupled device (CCD) camera (Wright Instruments, model 1, resolution 600 × 400 pixels, 14 bit) with a 50 mm Olympus macro lens. The technique has been described previously in studies on AlSPc, also in normal rat colon (Bedwell *et al.*, 1991). Strips of 4 cm length were taken from five animals, cut longitudinally, opened and placed flat onto glass slides for imaging; two animals received 200 mg $kg^{-1}$ ALA i.v. and a 50 J light dose at 4 h post-administration (as described below), and were immediately sacrificed with the 4 cm sections taken from the laser-treated area; two animals received the same ALA dose but no light dose and were sacrificed at 4 h; one animal only received an injection of PBS in order to assess background fluorescence levels. The colon strips were snap frozen in liquid nitrogen and stored at − 20°C before imaging. Fluorescence was excited at 488 nm using a 1 mW Ar ion laser beam expanded uniformly over the specimen, and detected with a 10 nm bandpass filter centred at 633 nm corresponding to the main fluorescence band of PPIX and eliminating potential interference from reduced photoprotoporphyrin photoproducts. An IBM PC clone with a high resolution colour monitor controlled the camera operation and was used for digital image processing, display and storage. Fluorescence was digitally quantified by either line or box superimposition on areas of interest. *In vivo* fluorescence spectra were recorded using a Perkin-Elmer spectrofluorimeter (LS-50B) equipped with a bifurcated optical fibre bundle for remote fluorescence sensing.

### *Photodynamic therapy*

The light source used was a pulsed (12 kHz) copper vapour pumped dye laser (Oxford Lasers, Oxford, UK). The output was tuned to 630 nm and delivered via a 200 μm fibre passed through the colon wall and just touching the normal mucosa on the opposite side. The fibre was maintained at approximately 90° to the mucosal surface. Any faeces present were eased away from the irradiation zone. The rest of the abdominal viscera was shielded from forward light scatter by interposition of a piece of opaque paper. Only one site in the colon was treated in each animal, approximately 1 cm distal to the caecum.

In the first study we varied the drug and energy dose. Power output from the fibre tip was 100 mW and the total irradiation time varied from 100 to 2000 s giving a total energy delivery of 10–200 J per animal. After each treatment the power output was checked to make sure there had been no significant drop of power during treatment. The time interval before PDT after sensitisation with ALA depended on the drug doses used and varied from 1 to 4 h (Loh *et al.*, 1992). Irradiation was performed at the time of peak PPIX concentration. Animals with the lowest concentration of ALA (25 mg $kg^{-1}$ i.v.) were treated after 1 h, those given 50 mg $kg^{-1}$ after 1.5 h, those given 100 and 200 mg $kg^{-1}$ after 2 h and those with the highest concentration of 400 mg $kg^{-1}$, after 4 h. Three to five animals were treated for each combination of variables tested. Unsensitised control animals were irradiated using similar light doses to exclude thermal effects. Another group of animals received sensitiser only to exclude any microscopic or macroscopic effect owing to ALA alone. After treatment, animals were allowed to recover and kept in standard laboratory conditions until killed at 72 h when mucosal damage, when present, was at a maximum (Barr *et al.*, 1987*b*). At post mortem, 3 cm of the colon distal to the caecum was excised and opened along the mesenteric border for macroscopic inspection. The specimens were laid out on a piece of card and the area of the PDT-induced lesions determined by taking the longest (*a*) and the shortest diameter (*b*) of the lesions which were approximately elliptical and then calculating the area by the formula $\pi ab/4$ (Barr *et al.*, 1987b). A small number of representative specimens was subsequently fixed in formalin and prepared for conventional light microscopy to confirm the macroscopic findings.

In the second series of experiments, the efficacy of different ways of fractionating the light dose was assessed. A light dose of 25 J (100 mW, 250 s) and drug dose of 200 mg $kg^{-1}$ ALA were used as these were known to give a 13 $mm^2$ lesion in normal colon applying the light in a single fraction and that small increases in either of these values would give a considerable increase in lesion size. Initially, the light dose of 25 J was divided into two or five equal fractions, with intervals of 10, 50, 150 300 and 900 s between fractions. Next, the number of fractions was varied from 1 to 25 (25 J × 1, 12.5 J × 2, 5 J × 5, 2.5 J × 10, 1 J × 25) with a fixed interval of 50 s between fractions. Finally, only one interval was used but at different times during treatment so the light fractions were not all the same (5 J then 20 J, 12.5 J then 12.5 J, 20 J then 5 J and 25 J in one fraction).

To assess the effect of light fractionation at ALA doses closer to those used clinically, a small number of experiments was also done at ALA doses of 50 and 100 mg $kg^{-1}$ i.v. All animals were killed 72 h after laser treatment and the area of the PDT lesions measured at post mortem. Five animals were used for each set of values tested.

### Results

### *Threshold studies*

Lesions were seen in the treated colons three days after PDT as well defined, oval shaped, necrotic ulcers. The maximum and minimum diameter of necrosis was measured macroscopically in all specimens. In the limited number of specimens examined microscopically, there was always close correlation between the dimensions of the lesion as measured macroscopically and microscopically, and so for the remainder of the study, only macroscopic measurements were made. The area of necrosis was plotted as a function of the applied energy for each ALA dose and is shown in Figure 1. With the lowest drug dose of 25 mg $kg^{-1}$, there was no difference in lesion size compared with unsensitised control animals irradiated with the same light doses (50, 100 or 150 J). For higher drug doses, initially the photodynamic effect increased with increasing light dose, but then reached a plateau (Figure 1). The same data is plotted in Figure 2 to show the area of necrosis as a function of the administered dose of ALA. For the higher energies of 100 and 150 J, a significant increase of the lesion size over that seen in controls could be achieved if the ALA dose was 50 mg $kg^{-1}$ or more. For the lower energies, this required elevating the dose to 100 mg $kg^{-1}$ (75 and 50 J) or 200 mg $kg^{-1}$ (25 J).

Photodegradation of ALA-induced PPIX was studied using *ex vivo* fluorescence imaging. Figure 3 shows that with a 50 J light dose the PPIX fluorescence was reduced by a factor of 3 in the centre of the strip (corresponding to the laser fibre position) and bleaching was evident over an extent of about 1 cm. A fluoresence emission spectrum of the same strip (excitation wavelength 400 nm) was recorded *ex vivo*. The central laser-treated region was probed with the excitation beam from the fluorimeter which showed the appearance of a new band around 675 nm which was not present in untreated specimens. For comparison, the two fluorescence maxima observed with ALA-induced PPIX in untreated rat

**Figure 1** Area of necrosis *vs* irradiation energy in normal rat colon after PDT at five different doses of ALA. Each point represents the mean of measurements from five animals. Standard errors were < ± 20%.

**Figure 2** Area of necrosis in normal rat colon after PDT. Same data as in Figure 1 but redrawn to show the variation of damage *vs* the dose of ALA. Standard errors were < ± 20%.

colon were observed at 636 and 704 nm. An identical *in vivo* spectrum was also recorded from the exposed colon of an anaesthetised rat (200 mg $kg^{-1}$ ALA, 2 h i.v.). An excitation spectrum of untreated colon was recorded from 600 to 650 nm using detection at 700 nm which showed that peak PPIX fluorescence excitation efficiency occurs at 635 nm; at 630 nm the efficiency was only 10% lower, thus little should be gained by using 635 instead of 630 nm for PDT treatment.

### *Fractionated irradiation studies*

In the second part of these studies we examined the influence of fractionating the light dose. Table I shows the results using just two fractions of light, but varying the interval between fractions from 10 to 900 s. Table II shows the results of dividing the light dose into five equal fractions, but once again varying the interval between fractions from 10 to 900 s. Table III shows the results of varying the number of fractions from 1 to 25, with equal fractions and a fixed interval of 50 s between fractions. Table IV shows the results using just two fractions with an interval of 300 s with the same total light dose of 25 J, but varying the size of the first fraction from 5 to 20 J. Finally, Table V shows the results using lower doses of ALA (50 and 100 mg $kg^{-1}$) compared with those found using 200 mg $kg^{-1}$.

### Discussion

ALA is an attractive prospect as a photosensitising agent and as skin photosensitivity is limited to 1–2 days, there are few risks of serious toxicity (as it is found in so many mammalian cells) and it can be given by mouth. Nevertheless, the maximum bolus dose that can be tolerated by mouth in patients is about 60 mg $kg^{-1}$. Higher doses cause nausea with occasional vomiting and transient elevation of liver enzymes (Regula *et al.*, 1995). However, our clinical studies on tumours of the gastrointestinal tract have shown that even using 60 mg $kg^{-1}$, it is not possible to produce necrosis that is more than 1 mm deep (Regula *et al.*, 1995). In contrast, we achieved 8 mm depth of necrosis in transplanted tumours in the hamster pancreas (Regula *et al.*, 1994) using an oral dose of 400 mg $kg^{-1}$ ALA. These results indicate that the drug doses used clinically were too low. This may be improved when an intravenous preparation of ALA is available for clinical use, particularly as the dose required intravenously is likely to be about half that needed orally to achieve the same tissue levels of PPIX (Loh *et al.*, 1993*a*). However, the tissue levels may still be relatively low. The purpose of the present investigation was to study the thresholds for drug and light doses required to get a PDT effect in normal rat colon and to

**Figure 3** Fluorescence image of ALA-induced PPIX-sensitised normal rat colon with the mucosa facing after *in vivo* laser treatment to the central section, 50 J and 200 mg $kg^{-1}$ ALA. Fluorescence was excited at 488 nm and detected at 630 nm. The false colour scale is shown at the top (white represents high levels, red low levels of PPIX). The white bar corresponds to 1 cm.

**Table I** Area of necrosis produced in normal rat colon with a drug dose of 200 mg $kg^{-1}$ ALA i.v. and irradiation with a light dose of 25 J. The light dose was split once and the duration of the break varied from 10 s to 15 min

| Duration of interruption | None | 10 s | 50 s | 150 s | 5 min | 15 min |
|---|---|---|---|---|---|---|
| Area of necrosis ($mm^2$) | 13 ± 2 | 89 ± 23 | 53 ± 7 | 94 ± 13 | 70 ± 13 | 52 ± 13 |

**Table II** Area of necrosis produced in normal rat colon with a drug dose of 200 mg $kg^{-1}$ ALA i.v. and irradiation with a light dose of 25 J. The light dose was split into five equal fractions and the duration of the breaks varied from 10 s to 15 min

| Duration of interruptions | None | 10 s | 50 s | 150 s | 5 min | 15 min |
|---|---|---|---|---|---|---|
| Area of necrosis ($mm^2$) | 13 ± 2 | 19 ± 7 | 26 ± 8 | 50 ± 8 | 55 ± 8 | 54 ± 7 |

**Table III** Area of necrosis produced in normal rat colon with a drug dose of 200 mg $kg^{-1}$ ALA i.v. and irradiation with a light dose of 25 J. The total energy dose of 25 J was split into a different number of fractions (1–25). The duration of the breaks was constant at 50 s

| Number of fractions | 1 | 2 | 5 | 10 | 25 |
|---|---|---|---|---|---|
| Area of necrosis ($mm^2$) | 13 ± 2 | 53 ± 7 | 26 ± 8 | 49 ± 7 | 44 ± 8 |

**Table IV** Area of necrosis produced in normal rat colon with a drug dose of 200 mg $kg^{-1}$ ALA i.v. and irradiation with a light dose of 25 J. The light dose was split once for 5 min. The first fraction was varied from 5 to 25 J

| First energy dose applied (J) | 5 | 12.5 | 20 | 25 |
|---|---|---|---|---|
| Area of necrosis ($mm^2$) | 93 ± 14 | 70 ± 13 | 47 ± 10 | 13 ± 2 |

see if it was possible to enhance the PDT effect by varying the treatment conditions.

In the first series of experiments we studied the size of PDT lesions produced as a function of the light dose for a range of doses of ALA and also as a function of the dose of ALA for a range of light doses. As would be expected, in general, the lesion area increased with the delivered light dose. This was true for ALA doses of 50 mg $kg^{-1}$ or more, but with 25 mg $kg^{-1}$, the lesion was never larger than that seen in unsensitised control animals, suggesting that there is a threshold between these two doses. This loss in reciprocity between the light and drug dose is most likely because of photodegradation of the PPIX during treatment as shown in Figure 3 where most of the PPIX over a 1 cm zone at the treatment site has been photodegraded by a light dose of 50 J. Similar results have been obtained with AlSPc (Barr *et al.*, 1990; Bedwell *et al.*, 1991) although the threshold dose for AlSPc is much lower at about 0.5 mg $kg^{-1}$.

It is difficult to extrapolate directly from rats to humans to correlate absolute tissue levels of PPIX with the administered dose of ALA without chemical extraction data. However we do have fluorescence photometric data recorded using the same calibration conditions from frozen sections (Loh *et al.*, 1993*a*, 1993*b*; Regula *et al.*, 1995) of rat colon and human colonic biopsies which indicate that the maximum dose we used clinically (60 mg $kg^{-1}$ by mouth) is very close to the threshold level found in the present experiments. Therefore it is perhaps not surprising that the clinical effects were so superficial. Nevertheless, some PDT necrosis was seen in our clinical study, which suggests that it may not be necessary to raise the tissue levels of PPIX very much in order to get a much larger effect. This problem may be solved when an intravenous preparation of ALA is available for clinical use, but another possibility is to give an iron chelating agent in order to inhibit the conversion of PPIX to haem and thus temporarily raise the tissue levels of PPIX. We have already shown that the mucosal level of PPIX in the rat bladder can be doubled by simultaneous administration of the iron chelating agent 1,2-diethyl-3-hydroxypyridine-4-one (CP94) (Chang *et al.*, 1995), although this has not yet been tested clinically.

At the upper end of the range of ALA doses used here, doubling the dose of ALA from 200 to 400 mg $kg^{-1}$ only produced a marginal increase in the area of the mucosal lesion. The most likely explanation for this is that the enzyme systems in the synthetic chain from ALA to PPIX became saturated thus limiting PPIX synthesis (Pottier *et al.*, 1986), so there is no point in giving an ALA dose more than 100–200 mg $kg^{-1}$. Thus there seems to be a fairly narrow band of effective doses of ALA, in the range 50–200 mg $kg^{-1}$.

General comments can also be made on the range of useful light doses. For ALA doses greater than 25 mg $kg^{-1}$, the area of necrosis increases with higher light doses, but little is gained by increasing the light dose above 100 J (Figure 2). Some effect is produced with 25 J, but with the lower doses of ALA, at least 50 J is required to produce a lesion of worthwhile size. Thus the useful range of light doses in this experimental model is quite small, between 50 and 100 J. Naturally, the light doses required in any particular situation will depend on the geometry and on the organ being treated, but these results do suggest that there is nothing to be gained by large increases in the doses of light. It has been speculated that the use of polychromatic light over the 600–700 nm range might enhance PDT with ALA through the conco-

**Table V** Area of necrosis produced in normal rat colon with different drug doses (50, 100, 200 mg $kg^{-1}$ ALA i.v.) and irradiation with different light doses (50, 25 and 25 J respectively). The treatment was interrupted once for 5 min after half of the energy had been delivered

| | | |
|---|---|---|
| Drug dose 50 mg $kg^{-1}$ ALA i.v. | 50 J continuous | 2 × 25 J (5 min break) |
| Area of necrosis ($mm^2$) | 10 ± 4 | 70 ± 11 |
| Drug dose 100 mg $kg^{-1}$ ALA i.v. | 25 J continuous | 2 × 12.5 J (5 min break) |
| Area of necrosis ($mm^2$) | 8 ± 3 | 63 ± 11 |
| Drug dose 200 mg $kg^{-1}$ ALA i.v. | 25 J continuous | 2 × 12.5 J (5 min break) |
| Area of necrosis ($mm^2$) | 13 ± 2 | 70 ± 13 |

mitant excitation of photoactive photoproducts, but experimental confirmation is still awaited. The presence of such photoproducts is, however, evident from fluorescence excitation studies from previous studies and this work where a new fluorescence band was observed at 675 nm which is characteristic of PPIX photoproducts (Konig *et al.*, 1993). The use of 635 nm excitation for ALA-induced PPIX PDT has recently been advocated although our fluorescence excitation studies indicate that any improvement over 630 nm should be marginal unless absorption by other porphyrin photoproducts with slightly red-shifted absorption spectra becomes important. Adequate light delivery in clinical practice will require mapping of the target tissue to determine the true extent of the area to be treated and then careful choice of light delivery systems so that appropriate light doses can be delivered to all relevant areas. This will require good-quality definition of the extent of the pathology, by imaging or other techniques, and then careful treatment planning by medical physicists. However, even with good planning, treatment times can be quite long to deliver the magnitude of doses shown here to be the most appropriate.

The second part of the experimental work in this paper was designed to assess one way in which PDT effects might be enhanced using different light dose regimens. The technique studied was fractionation of the light dose. To maximise the chances of finding a useful effect, we used a fairly high drug dose (200 mg $kg^{-1}$) and a low net light dose of 25 J as from our earlier results, under these conditions, a small enhancement of the PDT effect would give a large increase in the lesion size.

Our results show that fractionating the light dose can markedly enhance the PDT effect. By dividing the light into two fractions, the PDT-induced ulcers were at least three times larger and, in the case of a 150 s interval, an average of five times larger in area (Table I); all of the observed increases above the control value were well in excess of variations from experimental error. The data in Table II (when five fractions were used) suggest that the effect increases slightly as the duration of the break between fractions increases, although in Table I (just two fractions) this trend is not seen. Table III shows that the use of two fractions divided by a 50 s interval appears to be optimum with five fractions being slightly less effective but still a factor of two above the control value. It appears therefore that as long as there is at least one break in treatment, the number of breaks is not crucial.

Our experiments using 25 J and fractionated light produced lesions similar in size to those seen with continuous light doses of more than 100 J. Clinically, this could mean that the light dose can be reduced by a factor of four, which would be a considerable improvement. In our first experiments with just two fractions, the length of the break between fractions had little effect on the result (Table I) although with five fractions the lesion size appeared to increase as the interval was increased from 10 to 150 s. Experiments with larger numbers of animals would be required to establish how important this effect is overall. It could be relevant if new PPIX synthesis during the interval is important, but this is unlikely as the interval durations studied here are short compared with the time required for PPIX synthesis (van der Veen *et al.*, 1994).

The most likely reason for the effect of fractionation is related to tissue oxygenation and vascular shut-down. (Star *et al.*, 1986). Oxygen is an essential component of the PDT effect (Bown *et al.*, 1986; Star *et al.*, 1986) and we propose that some vasoconstriction occurs during the first fraction which partly relaxes during the break, so permitting reoxygenation of the target area and making it more susceptible to PDT when the next light fraction is delivered. In effect, it may be that the rapid onset of vasoconstriction soon after the start of irradiation is actually protective owing to the induced hypoxia; fractionation may then allow recovery of normal oxygen tension. As vasoconstriction is progressive during and after light exposure, the timing of the break between fractions is likely to be important, but the optimum time will probably vary a lot depending on the tissue being treated, the concentration of photosensitiser, the irradiance of the therapeutic light and various other factors. Furthermore, at certain light doses vasoconstriction is fully reversible after irradiation is complete. Arteries and veins behave slightly differently: venular constriction is delayed compared with arteriolar constriction as shown in studies on normal rat cremaster muscle. (McMahon *et al.*, 1994). These workers also maintain that although tumour vessels are inherently more fragile than in normal tissue, for example the cremaster muscle, similar degrees of microvascular damage occur in tumours implanted in the cremaster. Thus on this basis, although we have not presented any data on tumour models, we may be confident that our results on normal colon will be of relevance to a colonic tumour model which is a study we intend to pursue. Recently, studies of ALA-induced PPIX vascular PDT effects have been reported in experimental tumours (van der Veen *et al.*, 1994) which demonstrated that the magnitude of vascular damage is lower than observed with exogenous photosensitisers under corresponding tumoricidal conditions. The mechanisms involved in PDT-induced vasoconstriction have been recently reviewed (Wieman and Fingar, 1992) where it is noted that experimental studies of ischaemic injury have shown that temporary interruption of the regional blood flow leads to the release of oxygen radicals upon reperfusion (Klausener, 1989). Such an effect may well be involved in the enhanced response with fractionated PDT treatment if reperfusion is a significant effect during the intervals between irradiation.

Previous studies have compared continuous wave and pulsed laser sources for PDT and showed that there is no difference in the effect with high repetition rates (1–10 kHz with pulse widths of 10–40 ns) although no PDT effect was seen with a flashlamp pumped dye laser delivering higher intensity pulses with a repetition rate of $<10$ Hz and pulses of 2–400 μs duration (Cowled *et al.*, 1984; Barr *et al.*, 1989; Ferrario *et al.*, 1991; Panjehpour *et al.*, 1993). This absence of a PDT response at lower repetition rates was attributed to saturation pumping of the sensitiser resulting in depopulation of the ground absorbing state. This transient bleaching effect however can only occur using high power pulsed excitation as opposed to fractionated irradiation. Recently, there have been two papers looking at the effect of dividing the light into two fractions. Van der Veen *et al.* (1994) showed that two light fractions 90 min apart enhanced the PDT effect in a rat tumour model, but as the total delivered energy was doubled when the light was fractionated, it is not clear whether they were observing the same effect as us. Pe *et al.* (1994) studied the destruction of a transplanted tumour in mice after PDT with haematoporphyrin oligomer photosensitisation comparing a single treatment for 20 min with two 30 min treatments separated by 1 h, the total light dose being the same for the two regimens. They found that the fractionated regimen considerably enhanced the PDT effect. These results are certainly consistent with ours. Foster *et al.* (1991) have also demonstrated, using modulated irradiation with a period of 30 s, that a markedly improved tumour response with Photofrin can be achieved; although in our studies modulation with a 50 s period also proved effective, the degree of enhancement was greatest using a fractionated protocol.

In conclusion, although the maximum tissue levels of PPIX achieved in patients using oral ALA are probably only at or just above the threshold level for producing any effect, our experimental results indicate once the threshold level is exceeded, the efficacy is much greater, which implies that a useful clinical response could yet be achieved. In order to increase the PPIX levels ALA could be given intravenously and/or by coadministering an iron-chelating agent. Alternatively, by fractionating the light dose, even with low PPIX levels present, a significant improvement in the therapeutic response may be achievable.

**Acknowledgements**
Dr Messmann was funded by the Olympus Corporation, Dr Mlkvy was funded by the Association for International Cancer Research, Ms Davies by DUSA Pharmaceuticals, Mississauga, Ontario and Professor Bown by the Imperial Cancer Research Fund.

**References**

BARR H, TRALAU CJ, BOULOS PB, MACROBERT AJ, TILLY R AND BOWN SG. (1987*a*). The contrasting mechanism of colonic collagen damage between photodynamic therapy and thermal injury. *Photochem. Photobiol.*, **46**, 795–800.

BARR H, TRALAU CJ, MACROBERT AJ, KRASNER N, BOULOS PB, CLARK CG AND BOWN SG. (1987*b*). Photodynamic therapy in the normal rat colon with phthalocyanine sensitisation. *Br. J. Cancer*, **56**, 111–118.

BARR H, BOULOS PB, MACROBERT AJ, TRALAU CJ, PHILLIPS D AND BOWN SG. (1989). Comparison of lasers for photodynamic therapy with a phthlocyanine photosensitizer. *Lasers Med Sci.*, **4**, 7–12.

BARR H, TRALAU CJ, BOULOS PB, MACROBERT AJ, LEWIS MR, PHILLIPS D AND BOWN S.G. (1990). Selective necrosis in experimental colon cancer using photodynamic therapy with phthalocyanine photosensitisation. *Gastroenterology*, **98**, 1532–1537.

BEDWELL J, CHATLANI PT, MACROBERT AJ, ROBERTS JE, BARR H, DILLON J AND BOWN SG. (1991). Enhanced tumour selectivity of photodynamic therapy in the rat colon using a radioprotective agent. *Photochem. Photobiol.*, **53**, 753–756.

BEDWELL J, MACROBERT AJ, PHILLIPS D AND BOWN SG. (1992). Fluorescence distribution and photodynamic effect of ALA-induced PPIX in the DMH rat colonic tumour model. *Br. J. Cancer*, **65**, 818–824.

BOWN SG. (1990). Photodynamic therapy to scientists and clinicians – one world or two? *J. Photochem. Photobiol.*, **B**, **6**, 1–12.

BOWN SG, TRALAU CJ, COLERIDGE SMITH PD, AKDEMIR D AND WIEMAN TJ. (1986). Photodynamic therapy with porphyrin and phthalocyanine sensitisation: quantitative studies in normal rat liver. *Br. J. Cancer*, **54**, 43–52.

CHANG SC, MACROBERT AJ, PORTER JB AND BOWN SG. (1995). The effect of 1,2-diethyl-3-hydroxypyridine-4-one on tissue build-up of protoporphyrin IX: a microscopic quantitative fluorescence study on rat urinary bladder. *SPIE*, **2371**, 322–326.

COWLED PA, GRACE JR AND FORBES IJ. (1984). Comparison of the efficacy of pulsed and continuous-wave red laser light in induction of phototoxicity by haematoporphyrin derivative. *Photochem. Photobiol.*, **39**, 115–117.

COWLED PA AND FORBES IJ. (1985). Photocytotoxicity in vivo of hematoporphyrin derivative components. *Cancer Lett.*, **28**, 111–118.

DOUGHERTY TJ, POTTER WR AND WEISHAUPT KR. (1984). The structure of active component of hematoporphyrin derivative. In *Porphyrin Localisation and Treatment of Tumours*, Doiron D and Gomer C (eds) p. 301. Alan R Liss: New York.

FERRARIO A, RUCKER N, RYTER SW, DOIRON DR, GOMER CJ. (1991). Direct comparison of *in vitro* and *in vivo* Photofrin-II mediated photosensitization using a pulsed KTP pumped dye laser and a continous wave argon ion pumped dye laser. *Lasers Med. Sci.*, **11**, 404–410.

FINGAR VH AND HENDERSON BW. (1987). Drug and light dose dependence of photodynamic therapy: a study of tumor and normal tissue respone. *Photochem. Photobiol.*, **46**, 837–841.

FOSTER TH, MURANT RS, BRYANT RG, KNOX RS, GIBSON SL AND HILF R. (1991). Oxygen consumption and diffusion effects in photodynamic therapy. *Radiat. Res.*, **126**, 296–303.

GIBSON SL AND HILF S. (1985). Interdependence of fluence, drug dose and oxygen on hematoporphyrin derivative induced photosensitization of tumor mitochondria: *Photochem. Photobiol.*, **42**, 367–373.

GOSSNER L, WITTKE H, WARZECHA A, ERNST H, SROKA R, HAHN EG AND ELL C. (1994). Dose-dependent destruction of human gastrointestinal neoplasms by photodynamic therapy: a quantitative pilot study in athymic nude mice. *Eur. J. Gastroenterol. Hepatol.*, **6**, 159–165.

GRANT WE, HOPPER C, MACROBERT AJ, SPEIGHT PM AND BOWN SG. (1993). Photodynamic therapy of oral cancer: photosensitisation with systemic aminolaevulinic acid. *Lancet*, **342**, 147–148.

KONIG K, SCHNECKENBURGER H, RUCK A AND STEINER R. (1993). *In vivo* photoproduct formation during PDT with ALA-induced endogenous porphyrins. *J. Photochem. Photobiol.*, **B**, **18**, 287–90.

HENDERSON BW, BELLNIER DA, ZIRING B AND DOUGHERTY TJ. (1983). Aspects of the cellular uptake and retention of hematoporphyrin derivative and their correlation with the biological response to PRT in vitro. In *Porphyrin Photosensitization*. Kessel D and Dougherty TJ (eds) pp. 129–138. Plenum Press: New York.

KENNEDY JC AND POTTIER RH. (1992). Endogenous protoporphyrin IX, a clinically useful photosensitizer for photodynamic therapy. *J. Photochem. Photobiol. B: Biol*, **14**: 275–292.

KESSEL D. (1985). Proposed structure of the tumor localising component of 'hematoporphyrin derivative'. In *Photodynamic Therapy of Tumours and other Diseases*. Jori G and Perria C (eds) p. I. Libreria Progett: Padua.

KLAUSENER JM, PATERSON IS AND KOBSIIK L. (1989). Oxygen-free radicals mediate ischaemia-induced lung injury. *Surgery*, **105**, 192–99.

LOH CS, BEDWELL J., MACROBERT AJ, KRASNER N, PHILLIPS D AND BOWN SG. (1992). Photodynamic therapy of the normal rat stomach: a comparative study between di-sulphonated aluminium phthalocyanine and 5-aminolaevulinic acid. *Br. J. Cancer*, **66**, 452–462.

LOH CS, MACROBERT AJ, BEDWELL J, REGULA J, KRASNER N AND BOWN SG. (1993*a*). Oral versus intravenous administration of 5-aminolavulinic acid for photodynamic therapy. *Br. J. Cancer*, **68**, 41–51.

LOH CS, VERNON DI, MACROBERT AJ, BEDWELL J, BOWN SG AND BROWN SB. (1993*b*). Endogenous porphyrin distribution induced by 5-aminolaevulinic acid in the tissue layers of the gastrointestinal tract. *J. Photochem. Photobiol. B: Biol*, **20**, 47–54.

MCMAHON KS, WIEMAN TJ, MOORE PH AND FINGAR VH. (1994). Effects of photodynamic therapy using mono-aspartyl chlorin e6 on vessel constriction, vessel leakage, and tumour response. *Cancer Res.*, **54**, 5374–79.

MALIK Z AND DJALDETTI M. (1979). 5-Aminolevulinic acid stimulation of porphyrin and hemoglobins synthesis by uninduced Friend erythroleukemic cells. *Cell Differ.*, **8**, 223–233.

MALIK Z AND LUGACI H. (1987). Destruction of erythroleukaemic cells by photoactivation of endogenous porphyrins. *Br. J. Cancer*, **56**, 589–595.

MARRIOTT J. (1968). Regulation of porphyrin synthesis. *Biochem. Soc. Symp.*, **28**, 61–74.

PANJEHPOUR M, OVERHOLT BF, DENOVO RC, PETERSON MG AND SNEED RE. (1993). Comparative study between pulsed and continuous wave lasers for Photofrin photodynamic therapy. *Lasers Surg. Med.*, **13**, 296–304.

PE BM, IKEDA H AND INOKUCHI T. (1994). Tumour destruction and proliferation kinetics following periodic, low power light, haematoporphyrin oligomers mediated photodynamic therapy in the mouse tongue. *Oral Oncol. Eur. J. Cancer*, **30**, 174–178.

POTTIER RH, CHOW YFA, LAPAINTE J-P, TRUSCOTT TG, KENNEDY JC AND BEINER LA. (1986). Non-invasive technique for obtaining fluorescence excitation spectra and emission spectra *in vivo*. *Photochem. Photobiol.*, **44**, 679.

REGULA J, RAVI B, BEDWELL J, MACROBERT AJ AND BOWN SG. (1994). Photodynamic therapy using 5-aminolaevulinic acid for experimental cancer – evidence for prolonged survival. *Br. J. Cancer*, **70**, 248–254.

REGULA J, MACROBERT AJ, GORCHEIN A, BUONACCORSI GA, THORPE SM, SPENCER GM, HATFIELD ARW AND BOWN SG. (1995). Photosensitisation and photodynamic therapy of oesophageal, duodenal and colorectal tumours using 5-aminolaevulinic acid induced protoporphyrin IX. *Gut*, **36**, 67–75.

RIMINGTON C. (1966). Porphyrin and haem biosynthesis and its control. *Acta. Med. Scand.*, **179** (suppl 445), 11–45.

SIMA AAF, KENNEDY JC, BALKESLEE D AND ROBERTSON DM. (1981). Experimental porphyric neuropathy: a preliminary report. *Can. J. Neurol. Sci.*, **8**, 105–114.

STAR WM, MARIJNISSEN HPA, VAN DEN BERG-BLOK AE, VERSTEEG JAC, FRANKEN KAP AND REINHOLD HS. (1986). Destruction of rat mammary tumor and normal tissue microcirculation by hematoporphyrin derivative photoradiation observed *in vivo* sandwich observation chambers. *Cancer Res.*, **46**, 2532–2540.

VAN DER VEEN N, VAN LEENGOED HLLM AND STAR WM. (1994). *In vivo* fluoresence kinetics and photodynamic therapy using 5-aminolaevulinic acid-induced prophyrin: increased damage after multiple irradiations. *Br. J. Cancer*, **70**, 867–872.

WEISHAUPT KR, GOMER CJ AND DOUGHERTY TJ. (1976). Identification of singlet oxygen as the cytotoxic agent in the photoactivation of a murine tumour. *Cancer Res.*, **36**, 2326–2329.

WIEMAN TJ AND FINGAR VH. (1992). Photodynamic therapy. *Surg. Clin. N. Am.*, **72**, 609–621.